# Tandem lesions associate with angiographic progression of coronary artery stenoses

**DOI:** 10.1016/j.ijcha.2024.101417

**Published:** 2024-05-03

**Authors:** Kyle B. Franke, Nicholas J. Montarello, Adam J. Nelson, Jessica A. Marathe, Dennis T.L. Wong, Rosanna Tavella, Margaret Arstall, Christopher Zeitz, Matthew I. Worthley, John F. Beltrame, Peter J. Psaltis

**Affiliations:** a Adelaide Medical School, The University of Adelaide, Adelaide, Australia; b Lifelong Health Theme, South Australian Health and Medical Research Institute, Adelaide, Australia; c Department of Cardiology, Central Adelaide Local Health Network, Adelaide, Australia; d Victorian Heart Hospital, Clayton, Victoria, Australia; e Department of Cardiology, Northern Adelaide Local Health Network, Adelaide, Australia

**Keywords:** Atherosclerosis, Coronary artery disease, Quantitative coronary angiography, Plaque progression

## Abstract

**Background:**

Although the clinical factors associated with progression of coronary artery disease have been well studied, the angiographic predictors are less defined.

**Objectives:**

Our objective was to study the clinical and angiographic factors that associate with progression of coronary artery stenoses.

**Methods:**

We conducted a retrospective analysis of consecutive patients undergoing multiple, clinically indicated invasive coronary angiograms with an interval greater than 6 months, between January 2013 and December 2016. Lesion segments were analysed using Quantitative Coronary Angiography (QCA) if a stenosis ≥ 20 % was identified on either angiogram. Stenosis progression was defined as an increase ≥ 10 % in stenosis severity, with progressor groups analysed on both patient and lesion levels. Mixed-effects regression analyses were performed to evaluate factors associated with progression of individual stenoses.

**Results:**

199 patients were included with 881 lesions analysed. 108 (54.3 %) patients and 186 (21.1 %) stenoses were classified as progressors. The median age was 65 years (IQR 56–73) and the median interval between angiograms was 2.1 years (IQR 1.2–3.0). On a patient level, age, number of lesions and presence of multivessel disease at baseline were each associated with progressor status. On a lesion level, presence of a stenosis downstream (OR 3.07, 95 % CI 2.04–4.63, *p* < 0.001) and circumflex artery stenosis location (OR 1.81, 95 % CI 1.21–2.7, *p* = 0.004) were associated with progressor status. Other lesion characteristics did not significantly impact progressor status or change in stenosis severity.

**Conclusion:**

Coronary lesions which have a downstream stenosis may be at increased risk of stenosis progression. Further research into the mechanistic basis of this finding is required, along with its implications for plaque vulnerability and clinical outcomes.

## Highlights

• Our study has identified that the presence of downstream atherosclerotic disease is associated with worsening of more proximal stenoses over time.
• Further research into the effect of plaque morphology, geometry, PSS, ESS, and tandem stenoses on the natural history of coronary lesions is required.
• If a relationship between tandem stenoses and plaque progression is validated, it may have important implications for the revascularisation of coronary stenoses when downstream or upstream stenoses are present.

## 1 Introduction

Coronary atherosclerosis has a complex pathogenesis and is a leading cause of cardiovascular mortality, morbidity and health economic burden worldwide [1], [2]. While invasive coronary angiography (ICA) remains the gold standard for determining the presence of obstructive luminal stenoses in clinical practice [3], the use of coronary plaque imaging with intravascular ultrasound (IVUS), optical coherence tomography (OCT) or non-invasive computer tomography coronary angiography (CTCA) has allowed for more detailed and granular evaluation of plaque burden and composition, including their natural history and responsiveness to different treatments [4], [5], [6], [7], [8]. Despite this, IVUS and OCT remain expensive, carry added risks, and are not routinely performed during angiography, especially in the absence of percutaneous coronary intervention (PCI). This makes it difficult to use these modalities to survey a patient’s entire coronary vasculature and their risk of future ischaemic events in real-world clinical practice.

Studies that have relied on information from ICA itself have shown that the burden of coronary stenoses, including the presence of multivessel disease, associates with increased risk of future major adverse cardiovascular events [9], [10], [11]. In addition, other angiographic factors, such as degree of luminal stenosis and presence of lesion eccentricity, calcification and thrombosis may also be linked to the risk of acute coronary syndrome (ACS) [12]. Finally, growth of coronary plaques over time, as indicated by increased angiographic narrowing has also been shown to be a harbinger of plaque rupture and ACS [9], [10]. This suggests that progression of luminal stenosis as observed over multiple time-points with ICA may be a predictor of future ACS events. Therefore, the current study used quantitative coronary angiography (QCA) to analyse lesions found on serial angiograms and determine the clinical and angiographic characteristics that associate with progression of coronary stenoses over time.

## 2 Methods

### 2.1 Study design and setting

This was a retrospective analysis of successive patients included in the Coronary Angiogram Database of South Australia (CADOSA), established in 2012 to provide comprehensive data on invasive coronary angiography and patient-related outcomes, who had undergone one or more clinically indicated coronary angiograms in a single tertiary hospital, greater than six months from an index procedure performed between the 1st of January 2012 and the 31st of December 2016.

### 2.2 Patient selection

**Fig. 1:**
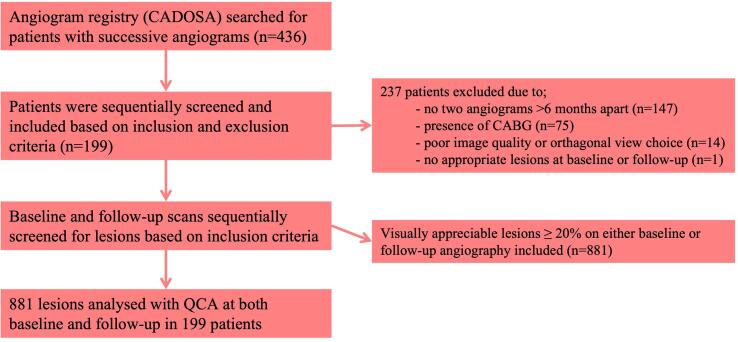
**Flow diagram for patient and lesion selection.** Flow diagram of the selection of patients and lesions for analysis in the study, including reasons for exclusion.

In patients in whom more than two coronary angiograms were performed, the two studies furthest apart were selected for analysis provided that no PCI had taken place in the intervening period. Patients with prior coronary artery bypass grafting (CABG) were excluded. Patients were also excluded if there were no coronary lesions causing ≥ 20 % stenosis at baseline or follow-up, inadequate image quality to perform QCA, or if there were limited images with appropriate orthogonal views at either time-point (Fig. 1). Ethical approval for waiver of consent for retrospective collection of clinical and angiographic patient data was obtained from our institution’s Human Research Ethics Committee (HREC/17/RAH/482).

### 2.3 Lesion selection and analysis

Baseline and follow-up angiograms were reviewed visually and any lesion with ≥ 20 % stenosis on either study was included in the analysis. The same coronary artery segment was analysed at both time points with the same or similar angiographic projection, with QCA analysis selected for the same length of lesion at both baseline and follow-up studies. Where PCI was performed as part of the baseline angiogram, stented lesions were excluded from the analysis. Where PCI was performed as part of the follow-up study, angiographic images prior to intervention were used in the analysis, if orthogonal view choice allowed for this. All stenoses were analysed by IntelliSpace Cardiovascular’s inbuilt QCA analysis software (Philips, version X). Reported QCA diameter was extracted from the “% Diameter Stenosis” variable from the QCA software and vessel calibre was estimated using the catheter calibration function. When required, correction of automatic contours was applied. Frame selection for QCA included selecting frames that were maximally opacified with no overlapping structures, with minimal vessel movement and frames that were as close to end-diastole as possible based on: (i) ECG-gating, (ii) catheter contrast-guided appearance, or (iii) visual assessment of myocardial contraction, in decreasing preferential order to limit variability and maximise precision and accuracy of QCA measurements [13].

### 2.4 Clinical variables

Clinical and laboratory data were collected from medical records. Blood results at the time of index angiography were preferentially used. Alternatively, results within a 4-week period of the index angiogram were used. When available, lipid panels were collected from both baseline and follow-up angiography. Estimated glomerular filtration rate (eGFR) was divided into two groups, <30 mL/min/1.73 m^2^ and ≥ 30 mL/min/1.73 m^2^.

### 2.5 Angiographic variables

American Heart Association criteria for angiographic lesion classification were employed with lesions categorised as (A) eccentric, (B) angulated, (C) bifurcation, or (D) diffuse [14]. These classifications were not mutually exclusive. Lesions were considered eccentric if one edge was at least three times narrower than the other when compared to the adjacent angiographically normal lumen [15]. Lesions that were > 20 mm in length were considered diffuse [14]. Lesion angle was defined as the direction of the artery at the beginning of the lesion compared to the end, with a lesion angle > 45° characterised as being angulated [14]. Bifurcation lesions were defined as lesions that occurred across a bifurcation of another artery that was at least 50 % of the calibre of the diseased artery. Lesions were considered to have downstream lesions if a second lesion ≥ 20 % stenotic was present more distally in the same artery or in a branch artery at least 50 % of the calibre of the diseased artery. Similarly, all lesions with a proximal stenosis ≥ 20 % were defined as having a lesion upstream. Lesions within the same coronary segment were considered distinct if they were separated by an angiographically normal coronary artery [16]. Multivessel disease was defined as the presence of a QCA stenosis > 50 % in at least two major epicardial coronary arteries or their major branches [16].

### 2.6 Statistical methods

All clinical and angiographic data were analysed using STATA15 (StataCorp LLC, College Station, TX). Data were reported in tables as ‘number, (percentage)’ [n, (%)] for categorical variables and either mean ± standard deviation (SD) or median [interquartile range (Q1,Q3)] for continuous variables, as appropriate. The normality of the variables was assessed using the Shapiro-Wilk test. All stenoses were given a ΔQCA% variable based on (follow-up QCA% – baseline QCA%).

In total, 45 lesions were analysed twice to calculate intra-observer variability, allowing for adequate power and comparison with previous research [17], [18]. The operator was blinded to the repeat analysis. Intra-observer variability was calculated with the intra-class correlation coefficient (ICC), including 95 % confidence intervals (95 %CI). Overall, intra-observer variability of QCA measurement was low (intra-class correlation coefficient 0.948, 95 %CI 0.902–0.972, mean difference 3.8 ± 3.6 %, range 0–12 %, n = 45). Only one analyst undertook QCA analysis, therefore analysis of inter-observer variability was not performed.

On a patient level, patients were divided into two groups; (i) “non-progressors” and (ii) “progressors” based on their largest ΔQCA% stenosis. Patients with their largest ΔQCA% < +10 % were defined as “non-progressors”, and those with their largest ΔQCA% ≥ +10 % were defined as “progressors”. These definitions were used to allow for intra-observer variability at both baseline and follow-up, while also intending to capture clinically meaningful stenosis progression. The differences in baseline clinical characteristics between these groups were then analysed with a chi-squared test for categorical baseline characteristics and a one-way ANOVA test for continuous baseline characteristics.

**Fig. 2:**
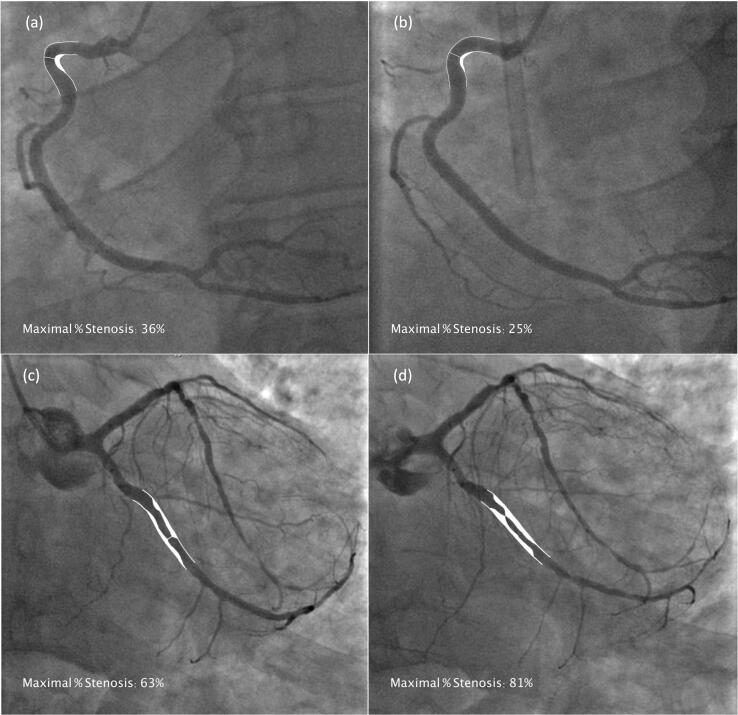
**Non-progressing and progressing lesions with QCA analysis overlay.** Examples of angiographic images with overlaid QCA results for maximal % stenosis, showing lesions in the proximal right coronary artery which did not progress from (a) baseline to (b) follow-up, and the left circumflex artery which did progress from (c) baseline to (d) follow-up.

On a lesion level, all stenoses were similarly categorised into “non-progressing stenoses” (ΔQCA% < +10 %), and “progressing stenoses” (ΔQCA% ≥ +10 %). Analysis of clinical and angiographic differences between these groups was then performed with a one-way ANOVA test for normal continuous characteristics, a Kruskal-Wallis test for non-normal continuous characteristics, and a chi-squared test for categorical characteristics. Fig. 2 demonstrates examples of QCA analysis for (a) non-progressing and (b) progressing stenoses.

Using logistic regression, univariate analyses were performed of both clinical and angiographic factors with progressor stenosis status as the outcome variable to create odds ratios (OR). Significant clinical and angiographic factors were then used in a multivariate analysis to predict progressor stenosis status. Using mixed-effects model regression, univariate and multivariate analyses were performed using the *xtmixed* function with the ΔQCA% variable. Univariate regression was performed on all clinical and angiographic characteristics (Model I). Multivariate analysis was performed on variables that were significant on univariate analysis (stepwise, *p* < 0.05), while those variables of significant clinical importance were forced into the model (Model II). Significant angiographic and relevant clinical characteristics were used for a fully adjusted model for angiographic characteristics (Model III).

## 3 Results

### 3.1 Baseline characteristics

Of the 436 patients identified who had undergone multiple coronary angiograms within the study window, 199 met all inclusion criteria and were ultimately included for analysis (Fig. 1). These patients had a total of 881 separate angiographic stenoses that were analysed at both time points (4.4 ± 2.3 stenoses per patient). Median age at the time of the first angiogram was 65 years [56, 73] and 144 (72.4 %) patients were male. Median interval between angiograms was 2.1 years [1.2, 3.0]. Ninety-one (45.7 %) patients were ultimately classified as non-progressors and 108 (54.3 %) as progressors. Acute coronary syndrome was the indication for angiography in 63 (31.7 %) patients at baseline and 47 (23.6 %) at follow-up.

### 3.2 Baseline clinical predictors of patient-level stenosis progression

Clinical factors that differed at baseline between progressors and non-progressors were age, the number of stenoses analysed, the most severe % stenosis at baseline, the most severe % stenosis at follow-up, the presence of multivessel disease at baseline and baseline SYNTAX score (Table 1). Specifically, progressors had a higher median age compared with non-progressors (66 [58, 75] vs 63 [53, 71], *p* = 0.042), greater severity of their worst stenosis at baseline (53 % [45, 64] vs 48 % [40, 59], *p* = 0.013) and follow-up (61 % [52, 75] vs 42 % [34. 51], *p* < 0.001), and more total stenoses analysed (4 [2,6] vs 3 [2,4], *p* < 0.001). Patients with multivessel disease at baseline were 3.6 times more likely to have progressor status (OR 3.6, 95 %CI 1.3–11.4, *p* = 0.005). Other clinical variables, including length of time between angiograms, gender, body mass index (BMI), hypercholesterolaemia, hypertension, smoking status, medications prescribed at baseline, baseline lipid levels and change in lipid levels from baseline to follow-up were not significantly different between groups.

**Table 1:** Clinical characteristics of patients by progressor status.

**Characteristic**	**All patients** **(n = 199)**	**Non-progressors (n = 91)**	**Progressors** **(n = 108)**	***p* value**
Age at baseline, years	65 [56, 73]	63 [53, 71]	66 [58, 75]	**0.042**
Follow-up, days	756 [443, 1091]	722 [438, 1091]	767 [480, 1098]	0.769
Number of stenoses analysed	4 [3,6]	3 [2,4]	4 [2,6]	**<0.001**
Most severe QCA stenosis at baseline (%)	51 [42, 62]	48 [40, 59]	53 [45, 64]	**0.013**
Most severe QCA stenosis at follow-up (%)	52 [41, 62]	42 [34, 51]	61 [52, 74.5]	**<0.001**
MVD at baseline, n (%)	28 (14.1)	6 (6.6)	22 (20.4)	**0.005**
Male, n (%)	144 (72.4)	62 (68.1)	82 (75.9)	0.221
BMI, kg/m^2^	28 [25, 33]	28 [25, 34]	28 [25, 32]	0.352
**Baseline Presentation**				
ACS at baseline, n (%)	63 (31.7)	29 (31.9)	34 (31.5)	0.953
LVEF (%)	59 [50, 66]	58 [50, 66]	59 [50, 68]	0.565
SYNTAX score	2 [0, 5]	0 [0, 3]	2 [0, 6]	**0.017**
**Medical History**				
Hypertension, n (%)	123 (61.8)	55 (60.4)	68 (63.0)	0.715
Diabetes, n (%)	71 (35.7)	32 (35.1)	39 (36.1)	0.850
Hypercholesterolaemia, n (%)	117 (58.8)	56 (61.5)	61 (56.5)	0.470
Smoker, n (%)	81 (40.7)	33 (36.3)	48 (44.4)	0.242
Previous MI, n (%)	45 (22.6)	19 (20.9)	26 (24.1)	0.591
Cardiomyopathy, n (%)	26 (13.1)	10 (11.0)	16 (14.8)	0.425
COPD, n (%)	25 (12.6)	7 (7.7)	18 (16.7)	0.057
Previous CVA/TIA, n (%)	12 (6.0)	5 (5.5)	7 (6.5)	0.771
eGFR < 30 mL/min/1.73 m^2^, n (%)	13 (7.0)	4 (4.4)	9 (8.3)	0.263
Dialysis, n (%)	11 (5.5)	4 (4.4)	7 (6.5)	0.521
**Medications Baseline**				
Anti-platelets, n (%)	181 (91.0)	80 (87.9)	101 (94.5)	0.170
Statin, n (%)	168 (84.4)	73 (80.2)	95 (88.0)	0.133
Ezetimibe, n (%)	13 (6.5)	9 (9.9)	4 (3.7)	0.079
ACEi/ARB, n (%)	164 (82.4)	88 (96.7)	76 (70.4)	0.707
Beta-blocker, n (%)	137 (68.8)	58 (63.7)	79 (73.1)	0.153
**Lipid levels Baseline**	n = 183	n = 84	n = 99	
LDL-Cholesterol (mmol/L)	2.3 [1.6, 3.1]	2.4 [1.8, 3.1]	2.2 [1.6, 3.2]	0.569
HDL-Cholesterol (mmol/L)	1.1 [0.9, 1.3]	1.1 [0.9, 1.3]	1.0 [0.9, 1.3]	0.657
Triglycerides (mmol/L)	1.6 [1.1, 2.4]	1.7 [1.1, 2.2]	1.6 [1.1, 2.6]	0.933
Total-Cholesterol (mmol/L)	4.2 [3.5, 5.2]	4.2 [3.6, 5.1]	4.2 [3.5, 5.2]	0.767
**Lipid levels Follow-up**	n = 117	n = 51	n = 66	
LDL-Cholesterol (mmol/L)	2.0 [1.5, 2.5]	2.0 [1.4, 2.5]	2.0 [1.6, 2.5]	0.476
HDL-Cholesterol (mmol/L)	1.0 [0.9, 1.2]	1.0 [0.9, 1.2]	1.0 [0.8, 1.2]	0.664
Triglycerides (mmol/L)	1.6 [1.1, 2.0]	1.6 [1.0, 2.2]	1.5 [1.1, 2.0]	0.883
Total-Cholesterol (mmol/L)	3.8 [3.2, 4.4]	3.6 [3.0, 4.4]	3.8 [3.3, 4.4]	0.383

Abbreviations: ACEi/ARB, angiotensin-converting enzyme inhibitor/angiotensin receptor blocker; ACS, acute coronary syndrome; BMI, body mass index; COPD, chronic obstructive pulmonary disease; CVA/TIA, cerebrovascular accident/transient ischaemic attack; eGFR, estimated glomerular filtration rate; HDL, high-density lipoprotein; LDL, low-density lipoprotein; LVEF, left ventricular ejection fraction; MI, myocardial infarction; MVD, multi-vessel disease; QCA, quantitative coronary angiography.

### 3.3 Clinical and angiographic predictors of stenosis progressor status

Table 2 describes the location and distribution of 881 stenoses analysed. Almost one-third (278, 31.6 %) were in the LAD, and one-quarter (213, 24.2 %) were in the RCA. Furthermore, the largest proportion of analysed lesions were in the mid-vessel (338, 38.4 %). Median baseline stenosis was 37 % [30–46] in the LAD, 36 % [28–47] in the circumflex, and 33 % [26–43] in the RCA.

**Table 2:** Baseline angiographic characteristics of stenoses.

**Angiographic Characteristic**	**All stenoses** **(n = 881)**	**Non-progressing stenoses (n = 695)**	**Progressing stenoses** **(n = 186)**	***p* value**
Artery involved Left main Left anterior descending Left circumflex Right Ramus intermedius Diagonal Obtuse marginal Other	26 (2.8)278 (31.5)178 (20.2)213 (24.2)7 (0.8)87 (9.9)71 (8.1)21 (2.4)	14 (2.2)229 (32.8)125 (18.0)171 (24.6)7 (1.0)73 (10.5)57 (8.2)19 (8.2)	12 (6.5)49 (26.3)53 (28.5)42 (22.6)0 (0.0)14 (7.5)14 (7.5)2 (1.1)	**0.001** 0.085 **0.002** 0.567 0.169 0.532 0.764 0.188
Segment of artery involved Proximal Middle Distal Side branch	222 (25.2)338 (38.4)142 (16.1)179 (20.3)	155 (22.3)256 (36.8)125 (18.0)149 (21.4)	67 (36.0)72 (38.7)17 (9.1)30 (16.1)	**<0.001** 0.913 **0.004** 0.110
Concentric vs eccentric stenosis Concentric Eccentric	563 (63.9)318 (36.1)	454 (65.3)241 (34.7)	109 (58.6)77 (41.4)	0.212
Focal vs diffuse narrowing Focal Diffuse	536 (60.8)345 (39.2)	412 (59.3)283 (40.7)	124 (66.7)62 (33.3)	0.067
Angulation of artery at stenosis 0-45° >45°	434 (49.3)447 (50.7)	345 (49.6)350 (50.4)	89 (47.8)97 (52.2)	0.664
Bifurcation stenosis	187 (21.2)	144 (20.7)	43 (23.1)	0.501
Baseline stenosis severity (%)	38 [29, 48]	39 [30, 48]	35 [25, 46]	**<0.001**
Presence of other downstream stenosis	385 (43.7)	258 (37.1)	127 (68.3)	**<0.001**
Presence of other upstream stenosis	475 (53.9)	398 (57.3)	77 (41.4)	**<0.001**

*Non-progressing stenoses were defined as < 10 % progression of QCA severity from baseline to follow-up and progressing stenoses were defined as those with ≥ 10 % progression. Data are displayed as number (n), percentage (%) for categorical variables and median [Q1,Q3] for continuous variables.

695 (78.9 %) stenoses did not progress from baseline to follow-up, while 186 (21.1 %) did. Clinical factors that associated with stenosis progressor status were male gender (OR 1.69, 95 %CI 1.11–2.57, *p* = 0.014) and eGFR < 30 mL/min/1.73.m^2^ (OR 2.09, 95 %CI 1.11–3.90, *p* = 0.022). Meanwhile, hypercholesterolaemia was negatively associated with stenosis progressor status (OR 0.70, 95 %CI 0.50–0.99, *p* = 0.046).

On univariate logistic regression analysis of angiographic factors, proximal segment location, circumflex and left main artery location and presence of a stenosis downstream were all associated with stenosis progressor status, whereas distal segment location and presence of a stenosis upstream were negatively associated with progressor status. After multivariate adjustment, angiographic factors that were significantly associated with progressor status were location in the circumflex artery (OR 1.81, 95 %CI 1.21–2.71, *p* = 0.004) and presence of a stenosis downstream (OR 3.07, 95 %CI 2.04–4.63, *p* < 0.001) (Table 3). Baseline stenosis severity was negatively associated with stenosis progression (OR 0.98, 95 %CI 0.97–1.00, *p* = 0.011).

**Table 3:** Clinical and angiographic predictors of stenosis progressor status.

**Variable**	**Model I**	***p* value**	**Model II**	***p* value**
**Clinical predictors**				
Age	[0.99–1.01]	0.816	1.00 [0.99–1.01]	0.836
Male gender	1.53 [1.04–2.26]	**0.031**	1.69 [1.11–2.57]	**0.014**
Hypertension	1.08 [0.78–1.51]	0.637	−	−
Diabetes Mellitus	1.00 [0.71–1.39]	0.987	−	−
Hypercholesterolaemia	0.72 [0.52–0.99]	**0.048**	0.70 [0.50–0.99]	**0.046**
Active smoking	1.26 [0.90–1.74]	0.168	−	−
Dialysis	1.74 [0.96–3.16]	0.069	−	**−**
eGFR < 30 mL/min/1.73 m^2^	1.60 [0.92–2.81]	**0.098**	2.09 [1.11–3.9]	**0.022**
Body Mass Index	1.00 [0.97–1.03]	0.762	−	−
Previous MI	1.12 [0.77–1.61]	0.552	−	−
ACS at baseline	0.95 [0.68–1.34]	0.788	−	−
Cardiomyopathy	1.26 [0.81–1.97]	0.300	−	−
LVEF	1.00 [0.98–1.01]	0.607	−	−
COPD	1.10 [0.68–1.77]	0.697	−	−
Previous CVA/TIA	1.09 [0.57–2.06]	0.802	−	−

**Angiographic predictors**				
Proximal location	1.96 [1.38–2.78]	**<0.001**	0.80 [0.50–1.26]	0.332
Distal location	0.46 [0.27–0.78]	**0.004**	0.94 [0.41–1.36]	0.332
Left circumflex artery	1.82 [1.25–2.64]	**0.002**	1.81 [1.21–2.71]	**0.004**
Left main artery	3.35 [1.52–7.38]	**0.003**	2.03 [0.86–4.80]	0.108
Eccentric stenosis	1.17 [0.89–1.53]	0.251	−	−
Diffuse stenosis	0.73 [0.52–1.02]	0.067	0.83 [0.58–1.19]	0.307
Angulated stenosis	1.07 [0.78–1.49]	0.664	−	−
Bifurcation stenosis	1.14 [0.78–1.69]	0.495	−	−
Baseline stenosis severity	0.98 [0.97–0.99]	**<0.001**	0.98 [0.97–1.00]	**0.011**
Stenosis downstream	3.62 [2.56–5.11]	**<0.001**	3.07 [2.04–4.63]	**<0.001**
Stenosis upstream	0.52 [0.38–0.72]	**<0.001**	0.85 [0.56–1.29]	0.456

Model I: Univariate analysis. Model II: Multivariate analysis adjusting for age, gender, hypercholesterolaemia, eGFR, and angiographic variables significant on univariate analysis. Abbreviations used: ACS, acute coronary syndrome; COPD, chronic obstructive pulmonary disease; CVA/TIA, cerebrovascular accident/transient ischaemic attack; eGFR, estimated glomerular filtration rate; LVEF, left ventricular ejection fraction; MI, myocardial infarction; MVD, multi-vessel disease.

### 3.4 Clinical and angiographic predictors of quantitative stenosis progression

The change in QCA% was + 0.7 ± 14.1 % across all 881 stenoses analysed. On mixed-effects regression analysis, male gender, hypercholesterolaemia and eGFR < 30 mL/min/.73 m^2^ were univariate predictors of stenosis progression (Table 4). After multivariate adjustment for age, gender, hypercholesterolaemia, smoking status and eGFR, male gender was associated with a 3.2 % increase in stenosis severity at follow-up (ΔQCA% +3.2 %, 95 %CI + 1.1 − +5.3 %, *p* = 0.003). Conversely, baseline hypercholesterolaemia was associated with a 2.7 % decrease in stenosis severity (ΔQCA% −2.7 %, 95 %CI −4.6 − −0.8 %, *p* = 0.006). An eGFR < 30 mL/min/1.73 m^2^ at baseline was associated with a 5.5 % increase in stenosis severity (ΔQCA% +5.5 %, 95 %CI + 1.8 − +9.2 %, *p* = 0.004). Active smoking showed a trend towards increased stenosis severity (ΔQCA% +1.7 %, 95 %CI −0.2 − +3.7 %, *p* = 0.084), whereas age did not have any effect.

**Table 4:** Mixed-effects regression analysis of clinical predictors of stenosis progression.

**Variable**	**Model I**	***p* value**	**Model II**	***p* value**
Age	0.0 % [-0.1, 0.1]	0.405	0.0 % [-0.1, 0.1]	0.999
Male gender	2.8 % [0.3, 5.4]	**0.028**	3.1 % [0.6, 5.6]	**0.014**
Hypertension	−0.6 % [-2.9, 1.8]	0.633	−	−
Diabetes Mellitus	0.3 % [-2.0, 2.7]	0.785	−	−
Hypercholesterolaemia	−2.5 % [-4.8, −0.2]	**0.034**	−2.4 % [-4.6, −0.2]	**0.033**
Active smoking	1.5 % [-0.8, 2.8]	0.213	1.7 % [-0.6, 4.0]	0.151
Dialysis	4.2 % [-0.7, 9.1]	0.092	−	−
eGFR < 30 mL/min/1.73 m^2^	4.7 % [0.2, 9.1]	**0.041**	6.0 % [1.5, 10.4]	**0.008**
Body Mass Index	0.1 % [-0.1, 0.3]	0.535	−	−
Previous MI	0.8 % [-1.8, 3.5]	0.536	−	−
ACS at baseline	−0.7 % [-3.2, 1.7]	0.550	−	−
Cardiomyopathy	0.3 % [-3.0, 3.6]	0.870	−	−
LVEF	0.0 % [-0.1, 0.1]	0.564	−	−
COPD	0.2 % [-3.2, 2.7]	0.897	−	−
Previous CVA/TIA	−1.7 % [-6.4, 3.0]	0.484	−	−

Model I: Univariate analysis. Model II: Multivariate analysis adjusting for age, gender, hypercholesterolaemia, smoking status, and eGFR. Abbreviations used: ACS, Acute Coronary Syndrome; COPD, chronic obstructive pulmonary disease; CVA, cerebrovascular accident; eGFR, estimated glomerular flow rate; LVEF, left ventricular ejection fraction; MI, myocardial infarction; TIA, transient ischaemic attack.

Univariate mixed-effects regression of angiographic lesion characteristics that associated with stenosis progression were significant for eccentricity, degree of baseline stenosis, presence of another stenosis downstream and presence of another stenosis upstream (Table 5). After adjusting for both clinical and angiographic factors, the degree of baseline stenosis was associated with a decrease in stenosis severity (ΔQCA% −0.3 %, 95 %CI −0.3 − −0.2 %, *p* < 0.001) and the presence of a stenosis downstream was associated with a 6.2 % increase in stenosis severity of the more proximal lesion (ΔQCA% +6.2 %, 95 %CI + 4.3 − +8.0 %, *p* < 0.001). Meanwhile, the presence of an upstream lesion only showed a trend towards decreased stenosis severity of the more distal lesion after multivariate adjustment (ΔQCA% −1.2 %, 95 %CI −3.0 − +0.7 %, *p* = 0.219).

**Table 5:** Mixed-effects regression analysis of angiographic predictors of stenosis progression.

	**Model I**	***p* value**	**Model II**	***p* value**	**Model III**	***p* value**
Eccentric stenosis	2.2 % [0.3, 4.1]	**0.022**	2.3 % [0.4, 4.2]	**0.018**	0.8 % [-1.0, 2.6]	0.382
Diffuse stenosis	−1.1 % [-3.0, 0.7]	0.238	−	−	−	−
Angulated stenosis	0.7 % [-1.1, 2.6]	0.445	−	−	−	−
Bifurcation stenosis	−0.8 % [-3.1, 1.4]	0.466	−	−	−	−
Baseline stenosis	−0.3 % [-0.3, −0.2]	**<0.001**	−0.3 % [-0.4, −0.2]	**<0.001**	−0.3 % [-0.3, −0.2]	**<0.001**
Stenosis downstream	8.0 % [6.3, 9.8]	**<0.001**	8.0 % [6.3, 9.7]	**<0.001**	6.2 % [4.3, 8.0]	**<0.001**
Stenosis upstream	−4.7 % [-6.5, −2.9]	**<0.001**	−4.7 % [-6.5, −2.9]	**<0.001**	−1.2 % [-3.0, 0.7]	0.219

Model I: Univariate analysis. Model II: Multivariate analysis adjusting for age, gender, hypercholesterolaemia, smoking status, and eGFR. Model III: Multivariate analysis adjusting for age, gender, hypercholesterolaemia, smoking status, eGFR, baseline QCA diameter, eccentric stenosis, calibre, and presence of tandem stenoses.

Finally, based on the finding that tandem lesions associated with progression of stenosis severity, we also compared the characteristics of those who had tandem stenoses with those who did not. A majority had at least one coronary artery containing tandem lesions (n = 168, 84.4 %), with only n = 31 (15.6 %) having no such lesions. Tandem stenoses were distributed evenly between the three major epicardial coronary arteries and were associated with: 235/278 (84.5 %) of LAD lesions, 150/178 (84.3 %) of circumflex lesions and 185/213 (86.9 %) of RCA lesions (p = 0.711). Acknowledging the small sample size of patients who had no tandem stenoses, we found that age was the only factor that significantly associated with the presence or absence of tandem lesions. Notably, patients who had tandem stenoses were no more likely to have diabetes, an ACS indication for the repeat angiogram or a longer time interval between angiographic studies (Supplementary Table 1).

## 4 Discussion

Consistent with previous findings linking plaque burden with increased progression of coronary atherosclerosis over time [19], our study found that the presence of multivessel disease and a higher number of stenoses were both associated with greater coronary stenosis progression, as defined by ΔQCA% analysis. Importantly, our results also demonstrate a novel interaction between tandem lesions, with proximal stenoses more likely to progress in the presence of distal disease. Among established cardiovascular risk factors, age, chronic kidney disease (eGFR < 30 mL/min/1.73 m^2^) and male gender were also associated with stenosis progression, whereas diabetes, smoking status, and hypertension were not.

The fact that modifiable cardiovascular risk factors did not impact stenosis progression may have been influenced by the relatively short time between angiograms analysed in the current study [20], and the introduction of risk factor-targeted treatments in those patients who had significant coronary disease at baseline angiography [3]. As a reflection of this, hypercholesterolaemia at baseline was associated negatively with patient-level progression. Although we speculate that those with documented hypercholesterolaemia at baseline may have received more aggressive lipid-lowering therapy to account for this finding, we found that neither baseline lipid levels nor change in lipid levels impacted the progress of coronary stenoses; however, it should be highlighted that only a limited number of patients had these data available. These results should therefore be interpreted in the context of a smaller sample size, as well as selection bias of those patients who were more likely to have lipids measured at both baseline and follow-up.

Our finding that the presence of a tandem lesion downstream was associated with progression of the more proximal stenosis is important. It is conceivable that this reflects biomechanical processes involved in atherogenesis and plaque progression, including the influence of endothelial shear stress (ESS) [21] and plaque structural stress (PSS) [22]. Endothelial shear stress is a measure of the tangential stress applied to the coronary artery wall due to coronary blood flow which influences endothelial function, smooth muscle cell behaviour, and inflammatory adhesion molecules [21]. Meanwhile, PSS is the mean of the maximal principal stress at the *peri*-luminal region. Increasingly, it seems likely that low ESS facilitates plaque initiation and progression, and that once developed, plaques subject to high PSS are at increased risk of developing vulnerable features [21], [22]. Interestingly, the results of our lesion-level multivariate analysis hint at a possible inverse relationship between the influence of upstream and downstream lesions on stenosis progression over time. We speculate that this is due to their different effects on ESS and/or PSS, and the changes in coronary blood flow and pressure that occur due to the haemodynamic influence of nearby stenoses. Few clinical trials or observational studies have explored the effect of sequential arterial stenoses; however, there are conceptual studies that have done so. Using computational fluid dynamics of sequential carotid stenoses, Li *et al.* evaluated the haemodynamic effects of tandem lesions [23]. The authors concluded that haemodynamic interactions between tandem stenoses are more complicated than the degree of luminal stenosis alone, with resultant PSS and ESS interactions altered by the geometric configuration of each stenosis. They demonstrated that areas of low ESS occur between two sequential stenoses, potentially leading to the progression of the upstream plaque, or the formation of a large continuous plaque, and cautioned that proximal stenosis endarterectomy or revascularisation may result in unfavourable haemodynamic conditions for the remaining distal stenosis. Clearly, further research on the influence of tandem plaques on coronary haemodynamic changes and their relationship with plaque progression is needed. The ongoing GEOMETRY trial, a prospective study of consecutive patients referred for clinically indicated CTCA should help to characterise the relationship between plaque geometry, endothelial stress and plaque burden and progression (NCT04185493) [24].

The observation that the circumflex artery was associated with progression of stenosis severity was unexpected, as to the best of our knowledge this has not been described previously. In a recent study by Bax *et al*., 1,344 low-risk patients underwent serial imaging by non-invasive CTCA with a median interval of 3.3 years between scans [25]. These authors found that the left circumflex artery had significantly smaller initial plaque volume at baseline, lower rates of progression of plaque volume and lower rates of high-risk plaque features such as positive remodelling, compared to the LAD and RCA. They concluded that this could either represent an earlier stage of atherosclerotic disease in the circumflex artery of these patients, or a different pathologic milieu between different epicardial coronary arteries. In contrast, our results showed similar stenosis severity at baseline across the three main coronary arteries, but greater stenosis progression in the circumflex. Our data does not point to a clear reason why this should have been the case, especially as there was not a higher prevalence of tandem lesions involving the circumflex system. Nevertheless, our results and those from the Bax study highlight that there may be differences in the natural history of plaque progression between different coronary vessels. This may have therapeutic implications for decision-making around coronary revascularisation and needs further studies to elucidate responsible mechanisms.

Limitations of our study include its small sample size and retrospective nature. We also had limited access to some patient data, such as paired lipid results which were not routinely available. Poor image quality at either baseline or follow-up resulted in patient exclusion and potentially resulted in selection bias. Further, there was uncertainty surrounding the use of intracoronary GTN prior to lesion evaluation by QCA in a small number of patients, potentially resulting in random error at a patient level. QCA is further limited by only assessing luminal stenoses and is thus unable to assess plaque composition or fully encapsulate the progression of lesions with positive remodelling, that is, progression of plaques towards the external elastic media rather than towards the lumen of the artery [26]. Other limitations of QCA include vessel motion limiting image quality, foreshortening of the coronary artery, and geometric patient variability. These limitations were minimised by assessing multiple orthogonal views for each stenosis analysed, selecting the same orthogonal views at baseline and follow-up for the respective lesion analysed, and optimal frame selection of the end-diastolic frame as described. Reliance on a single analyst was also a limitation; however, we attempted to overcome this by undertaking training sets prior to beginning formal analysis and allowing for intra-observer variability in the classification of both patient and stenosis progression groups. Furthermore, overall intra-observer variability was low compared with previous studies [17], [18].

In conclusion, based on QCA analysis of serial coronary angiograms, our study has identified that the presence of downstream atherosclerotic disease is associated with worsening of more proximal stenoses over time. Further research into the effect of plaque morphology, geometry, PSS, ESS, and tandem stenoses on the natural history of coronary lesions is required. Similarly, prospective studies utilising intracoronary imaging may shed more light on this relationship. If a relationship between tandem stenoses and plaque progression is validated, it may have important implications for the revascularisation of coronary stenoses when downstream or upstream stenoses are present.

## CRediT authorship contribution statement

**Kyle B. Franke:** Writing – original draft, Formal analysis, Data curation, Conceptualization. **Nicholas J. Montarello:** Writing – review & editing, Formal analysis, Data curation. **Adam J. Nelson:** Writing – review & editing. **Jessica A. Marathe:** Writing – review & editing. **Dennis T.L. Wong:** Writing – review & editing. **Rosanna Tavella:** Writing – review & editing, Funding acquisition, Data curation. **Margaret Arstall:** Writing – review & editing. **Christopher Zeitz:** Writing – review & editing. **Matthew I. Worthley:** Writing – review & editing, Conceptualization. **John F. Beltrame:** Writing – review & editing, Funding acquisition, Conceptualization. **Peter J. Psaltis:** .

## Declaration of competing interest

The authors declare that they have no known competing financial interests or personal relationships that could have appeared to influence the work reported in this paper.
