# Supplementary material for: Tandem lesions associate with angiographic progression of coronary artery stenoses
Source: Int J Cardiol Heart Vasc. 2024 May 3;52:101417. doi: 10.1016/j.ijcha.2024.101417 (PMC11079457; doi:10.1016/j.ijcha.2024.101417)
Supplement: Supplementary Data 1 [file mmc1.docx]

Franke et al. Supplementary Material

**Tandem lesions associate with angiographic progression of coronary artery stenoses**

Kyle B Franke^1^, Nicholas J Montarello^2,3^, Adam J Nelson^1,2,3^, Jessica A Marathe^2,3^, Dennis T L Wong^4^, Rosanna Tavella^1,3^, Margaret Arstall^15^, Christopher Zeitz^1,3^, Matthew I Worthley^1,2,3^, John F Beltrame^1, 3^, Peter J Psaltis^1,2,3^

^1^Adelaide Medical School, The University of Adelaide, Adelaide, Australia

^2^Lifelong Health Theme, South Australian Health and Medical Research Institute, Adelaide, Australia

^3^Department of Cardiology, Central Adelaide Local Health Network, Adelaide, Australia

^4^Victorian Heart Hospital, Clayton, Victoria, Australia

^5^Department of Cardiology, Northern Adelaide Local Health Network, Adelaide, Australia

**Short title:** Predictors of coronary plaque progression

*** Corresponding Author:**

Associate Professor Peter J. Psaltis

Heart and Vascular Program, Lifelong Health Theme

South Australian Health and Medical Research Institute

PO Box 11060, Adelaide, SA, 5001, Australia.

Tel: +61 8 8128 4534

Email: [peter.psaltis@sahmri.com](mailto:peter.psaltis@sahmri.com)

**Supplementary Table 1: Clinical characteristics of patients by tandem lesion status**

| **Characteristic** | **All patients (n=199)** | **Tandem lesions (n=168)** | **No tandem lesions (n=31)** | ***p* value** |
| --- | --- | --- | --- | --- |
| Age at baseline, years | 65 [56, 73] | 66 [58, 73] | 59 [50, 71] | **0.035** |
| Interval between angiograms, days | 756 [443, 1091] | 762 [442, 1116] | 691 [444, 930] | 0.449 |
| Number of stenoses analysed | 4 [3, 6] | 5 [3, 6] | 1 [1, 2] | **<0.001** |
| MVD at baseline, n (%) | 28 (14.1) | 27 (16.1) | 1 (3.2) | 0.059 |
| Male, n (%) | 144 (72.4) | 124 (73.8) | 20 (64.5) | 0.288 |
| BMI, kg/m^2^ | 28 [25, 33] | 28 [25,32] | 29 [25, 34] | 0.329 |
| Hypertension, n (%) | 123 (61.8) | 103 (61.3) | 20 (64.5) | 0.736 |
| Diabetes, n (%) | 71 (35.7) | 58 (34.5) | 13 (41.9) | 0.429 |
| Hypercholesterolaemia, n (%) | 117 (58.8) | 99 (58.9) | 18 (58.0) | 0.928 |
| Smoker, n (%) | 81 (40.7) | 70 (41.7) | 11 (35.5) | 0.520 |
| Previous MI, n (%) | 45 (22.6) | 42 (25.0) | 3 (9.7) | 0.061 |
| Reason for repeat angiography   - ACS - Other (Stable angina, valve work-up, etc) | - 47 (23.6) - 152 (76.4) | - 40 (23.8) - 128 (76.2) | - 7 (22.6) - 24 (77.4) | 0.882 |

Comparison of characteristics for patients divided into two groups based on the presence (n=168) or absence of tandem lesions (n=31). Abbreviations: ACS, acute coronary syndrome; BMI, body mass index; MI, myocardial infarction; MVD, multivessel disease. Patients were divided into two groups based on the presence of tandem lesions (n=168) or the absence of tandem lesions (n=31).
